# A compact SiGe D-band power amplifier for scalable photonic-enabled phased antenna arrays

**DOI:** 10.1038/s41598-023-47908-w

**Published:** 2023-11-23

**Authors:** Reinier Broucke, Nishant Singh, Michiel Van Osta, Joris Van Kerrebrouck, Piet Demeester, Sam Lemey, Guy Torfs

**Affiliations:** Department of Information Technology, IDLab, Ghent University - imec, 9052 Ghent, Belgium

**Keywords:** Electrical and electronic engineering, Silicon photonics

## Abstract

To address the rising demand for high-speed wireless data links, communication systems operating at frequencies beyond ${100}\,\hbox {GHz}$ are being targeted. A key enabling technology in the development of these wireless systems is the phased antenna array. Yet, the design and implementation of such steerable antenna arrays at frequencies over ${100}\,\hbox {GHz}$ comes with a multitude of challenges. In particular, the cointegration of active electronics at each antenna element poses a major hurdle due to the inherent space constraints in the array. This article proposes a novel scalable concept for opto-electronic phased antenna arrays operating at 140 GHz. It details the system architecture of a transmitter that enables the implementation of large scale, wideband, 2D steerable phased antenna arrays and presents the design and measurement of a compact SiGe power amplifier (PA) chip to be used as one of its key building blocks. The amplifier achieves a gain of 20 dB at 135 GHz, features a $P_{1dB}$ of 14.6 dBm and can support data rates up to 45 Gbps in a limited footprint of only 540μm × 550μm. This makes it one of the fastest, most powerful D-band power amplifiers in literature with a footprint compatible with $\frac{\lambda }{2}$-spaced phased array integration.

## Introduction

High-speed wireless communication has seen a significant increase in demand over the past few years. The development and subsequent rollout of 5G networks has a significant contribution to this. In order to support the increasing demand for wireless communication bandwidth, higher millimeter-wave (mmWave) and sub-THz carrier frequencies are under investigation^1–3^. Currently, the D-band (110 GHz – 170 GHz) is an attractive candidate, not only for high-speed wireless communication^4^ but also for applications like high-resolution radar front ends^5,6^ or spectroscopy^7^. This can be attributed to the very large aggregated available and unlicensed bandwidth. However, the realisation of such wireless D-band systems comes with many challenges. In particular, path loss experienced by the signal is a severely limiting factor in the performance of these mmWave systems, increasing by approximately 15 dB when moving from 5G mmWave at approximately 30 GHz to the D-band at 140 GHz. As such, to fully exploit the potential of wireless D-band communication links, technologies used in the 5G mobile communication network will need to evolve further, incorporating novel advancements and innovations to further enhance spectral efficiency, energy efficiency, and reliability. One key enabling technology that will be indispensable for these D-band systems are steerable or phased antenna arrays. These antenna arrays significantly increase link budget compared to a single antenna element, up to $20log_{10}(N)$ dB^8^, with N being the number of elements in the array. Note that this is only the case if every antenna is equipped with its own dedicated power amplifier, thereby also upscaling the transmitter’s power consumption. The total array gain can be further increased to $30log_{10}(N)$ if a similar array is used at the receiver side. This allows the signal to overcome the extreme propagation loss at D-band frequencies. However, this compensation requires a large amount of antennas, raising the need for an efficient, highly scalable solution. For instance, commercial mmWave antenna arrays typically use between 16 and 64 antennas for 5G wireless networks around 30 GHz^9,10^ and up to 256 antenna elements for 60 GHz fixed wireless access networks^11^. If this trend continues, it is safe to assume that commercially viable D-band arrays will use hundreds, if not thousands of antenna elements. Their steerability is not only a crucial feature in mobile systems with fast-moving users, but also for alignment purposes in fixed-beam use cases due to the narrow beamwidth of large-scale antenna arrays^12^.

The implementation of very large (phased) antenna arrays raises a number of challenges itself. First, there is the signal distribution problem. If we assume the case of purely passive splitting and routing, the maximum number of elements in the array is limited by the feeding losses. Expanding the array beyond a certain size has an adverse effect because the increase in array gain is negated by additional feeding losses. It is possible to overcome this limitation using additional amplification hardware as shown in^13^. Unfortunately, this significantly increases the complexity and power consumption of the circuit and is difficult to implement at such high frequencies. As such, an efficient signal distribution network is imperative to be able to support large antenna arrays. The steerability requirement of the array also raises the question of how to handle beamsteering. Traditional electronic beamsteering is implemented by controlling the phase shift between the different array elements. This narrowband approximation of a delay introduces beam squint, where the array’s beam is steered in different directions depending on the frequency of the signal^14^. Evidently, this can have drastic effects on performance when steering broadband signals over a large scanning range using an array with a very narrow beamwidth. Shifting to a time delay-based approach avoids this issue but is difficult to achieve electronically over wide delay spreads^15^. These particular challenges can be overcome by leveraging optical techniques. The inclusion of optical components is already prevalent in existing radio systems in the form of radio-over-fiber (RoF). Here, optical fiber infrastructure is used to distribute radio signals from a central office (CO) to remote radio units (RRUs)^16–18^. Moving to such a partially optical system allows us to leverage the wide gamut of optical components and techniques such as extremely low-loss signal distribution, true-time delay (TTD) beamforming or even on-chip optical modulation to overcome the issues faced in an all-electrical approach^19–21^.

Lastly, one of the most restricting issues in the design of steerable D-band arrays lies with the cointegration of active electronics with each individual antenna element. As shown by Sadhu et al.^12^, the unit elements in antenna arrays and their respective spacing shrink as the array’s frequency of operation increases. However, the active electronics driving each individual antenna element don’t shrink at nearly the same rate as the antennas do. In the past, this issue was much less prevalent since even at 30 GHz, a $\frac{\lambda }{2}$-spaced array still has an area of 25mm^2^ per antenna element to accomodate integrated circuits (ICs) for amplification, beamforming, digital control etc. The importance of this issue becomes clear when reviewing the current state-of-the-art on sub-THz phased antenna arrays in literature. Kim et al.^22^ and Gomez-Torrent et al.^23^ present the implementation of very large-scale, 2D arrays. While these achieve very good bandwidth and gain, they do not support beamsteering or integration of other active electronics due to their multilayer waveguide feeding network. Laminen et al.^24^ show a 4×4 D-band antenna array with a fractional bandwidth of 14%, although no beamsteering electronics are integrated and the high microstrip feedline losses limit scaling to larger antenna arrays. Other implementations are steerable, yet achieve this through varying the center frequency, giving rise to beam squint^25,26^. This imposes a significant data rate limit on the system. Abu-Surra et al.^15^ present a phased array with a substantial steering range, although this can only be done in one dimension over a limited 2 GHz bandwidth. Finally, Dong et al.^27^, Elkhouly et al.^28^ and Ahmed et al.^29^ all show very impressive implementations and demonstrations for steerable Tx/Rx arrays, with Ahmed et al. showing actual beamsteering measurement results. It should be noted however that scaling in all of these solutions is limited as they rely on extra tunable gain elements to compensate for high-frequency transmission line feeding losses. In summary, despite the impressive strides made in this research area in these past few years, the authors of this work currently have no knowledge of a solution that enables the implementation of very large-scale, wideband, 2D-steerable antenna arrays.

This work presents a novel, scalable concept for opto-electronic active phased antenna arrays operating in the D-band. The proposed system will provide a solution to the aforementioned issues currently plaguing the design of sub-THz phased antenna arrays. Most importantly, this work details the design and verification of a D-band power amplifier IC that suitable for integration into the aforementioned system. First, the Opto-Electronic Transmitter System Architecture section will discuss the proposed opto-electronic transmitter system architecture, briefly elaborate on the function of each component and derive specifications for the required power amplifier. Next, the Power Amplifier Design & Measurements section elaborates on the development of this power amplifier IC, including both the design procedure and measurement results. Lastly, the Conclusion section summarizes the main findings of this manuscript.

**Figure 1:**
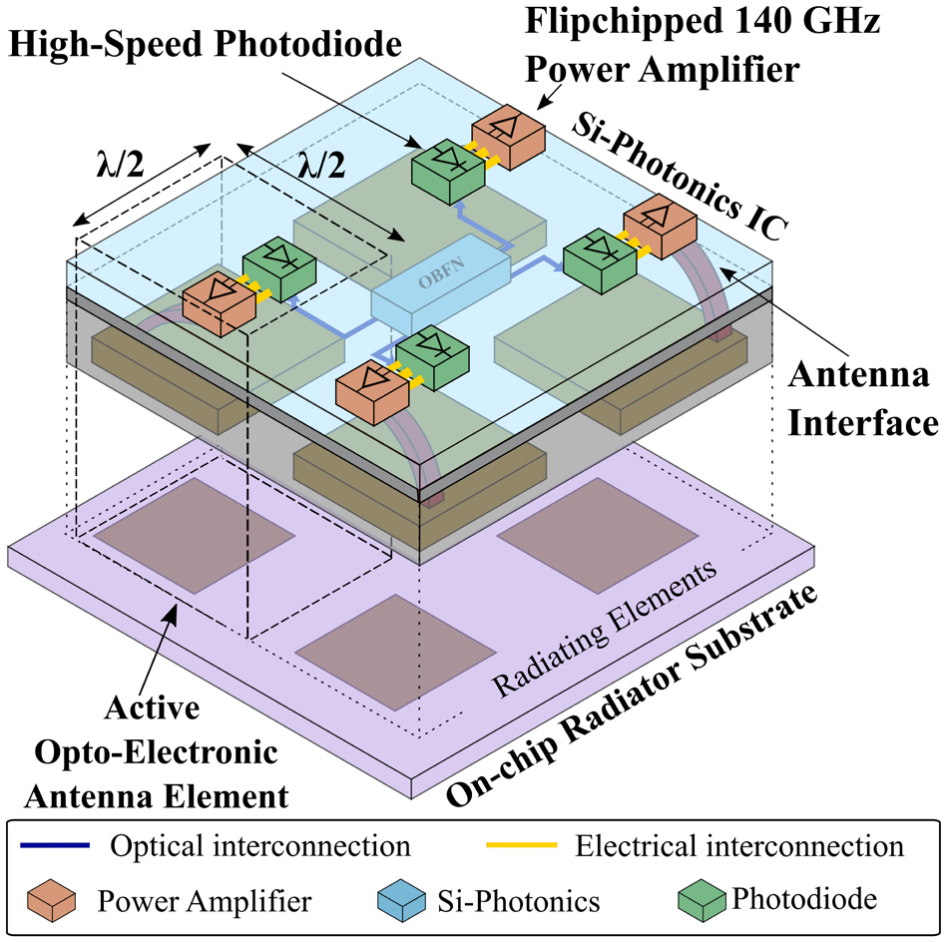
Schematic of the proposed opto-electronic phased antenna array.

## Opto-electronic transmitter system architecture

The introductory section already touched on some of the most significant challenges to be addressed in the development of D-band phased antenna arrays. These include low-loss signal distribution, broadband and beam squint-free beamsteering and finally, the cointegration of the array with active electronics.

To tackle these problems, we propose a novel concept for a D-band phased array transmitter. The block diagram of the proposed system architecture for this multi-antenna system, shown in Figure 1, represents the implementation of an exemplary 2×2 array. To alleviate the drawbacks of conventional all-electronic approaches, an opto-electronic architecture is adopted. The transmitter is implemented as an array of active opto-electronic antenna elements supported by an optical front-end module. The active antenna element implements opto-electric conversion, amplification and radiation. The optical front-end, which is implemented on a silicon-photonics (Si-Ph) PIC, handles the signal distribution and beamforming. Notice that, using this architecture, the system is not limited to a 4-element configuration and can be scaled up further to hundreds of antennas according to the specific application. The next paragraph explains the function and specifications of each component in more detail.

At 140 GHz, antenna elements suited for phased array integration are ideally around 1 mm × 1 mm (half of the free-space wavelength) in size with a spacing between 1 mm and 1.3 mm to ensure a grating lobe-free steering range of 180° down to 60° respectively^30^. This yields an integration density of approximately 100 antennas per cm^2^. To facilitate the broadband feeding of such a high number of densely packed antennas while enabling two-dimensional beamsteering, we propose integrating these antenna elements into the backside of the substrate of a photonic IC as is shown in Figure 1. This PIC itself is used as a platform for signal distribution as well as beamforming. By employing an analog radio-over-fiber (ARoF) scheme where the 140 GHz signal is directly modulated onto an optical carrier, the signal can be distributed over the array through ultra low loss optical waveguides. The loss of these waveguides can be as low as 3 dB/m^31^, compared to a loss of approximately 1 dB/cm for state-of-the-art electrical waveguides at these frequencies^32^. Because of this low loss, the PIC can easily be reused to implement an optical beamforming network (OBFN)^33,34^ through a network of switchable delay lines which act as true-time delay (TTD) cells. As these apply a different phase to each frequency of the signal, the issue of beam squint is negated. While the optical waveguides on the PIC offer low-loss interconnects to each of the antennas, these optical signals should still be converted into an electrical current with a high-speed photodiode (PD). At D-band frequencies, PDs can typically only supply a maximum of a few milliwatts of power^35^. Hence, a dedicated D-band power amplifier is required at each antenna element to provide amplification of the signal up to a level suitable for transmission. As mentioned in the introductory paragraph, the small spacing between the antenna elements severely restricts the footprint of the electronics that are integrated together with each antenna element. In turn, this has serious implications on the performance of the PA. Its footprint has to be limited to a fraction of the wavelength that corresponds to the highest frequency of operation, meaning that a system-level bandwidth specification should be derived. When considering a system-level point of view, a throughput of at least 100 Gbps is targeted to enable next-generation high-throughput applications. If we assume relatively simple modulation schemes such as 16-QAM or 32-QAM, a bandwidth of ${20}\,\hbox {GHz}$ to ${25}\,\hbox {GHz}$ is required. This is also in line with the current state-of-the-art for power amplifiers at this frequency^8,36,37^. If we consider a realistic steering range of [${-45}^\circ ,{45}^\circ$], the inter-element spacing cannot exceed $0.59\lambda _{min}$ × $0.59\lambda _{min}$ where $\lambda _{min}$ is the wavelength corresponding to the array’s highest frequency of operation^30^. Combined with a 20 GHz bandwidth centered around 140 GHz, the PA’s dimensions can certainly not exceed ${1.18}\,\hbox {mm}$ × ${1.18}\,\hbox {mm}$. However, considering that space should be left to accomodate PD integration and DC routing, a more stringent ${0.8}\,\hbox {mm}$ $\times$ ${0.8}\,\hbox {mm}$ footprint restriction will be imposed. This limits both the output power and the gain of the PA as both power combining techniques and the cascading of many amplifier stages are off the table.

## Power amplifier design & measurements

In this section, we will describe the design and validation of a compact D-band power amplifier. Because of the very high frequency of operation, a high-performance semiconductor process is a necessity to achieve acceptable gain and output linearity. If RF performance is the only concern, an InP or other III/V technology would be an obvious choice as these devices feature very high $f_{T}/f_{max}$ and good voltage handling, making them very attractive options for the development of sub-THz power amplifiers^38^. However, their larger size, restrictive back-end-of-line (BEOL) and lack of support for logic or control circuitry makes them less favorable than eg BiCMOS technologies^37,39,40^. In this work, a SiGe BiCMOS technology with $f_{T}/f_{max}$ of $350/{450}\,\hbox {GHz}$ and a copper BEOL was used. The latter is crucial as it enables low-loss interconnections and passives. The following paragraphs elaborate further on both the design and measurement results of this power amplifier chip.

### Circuit implementation

**Figure 2:**
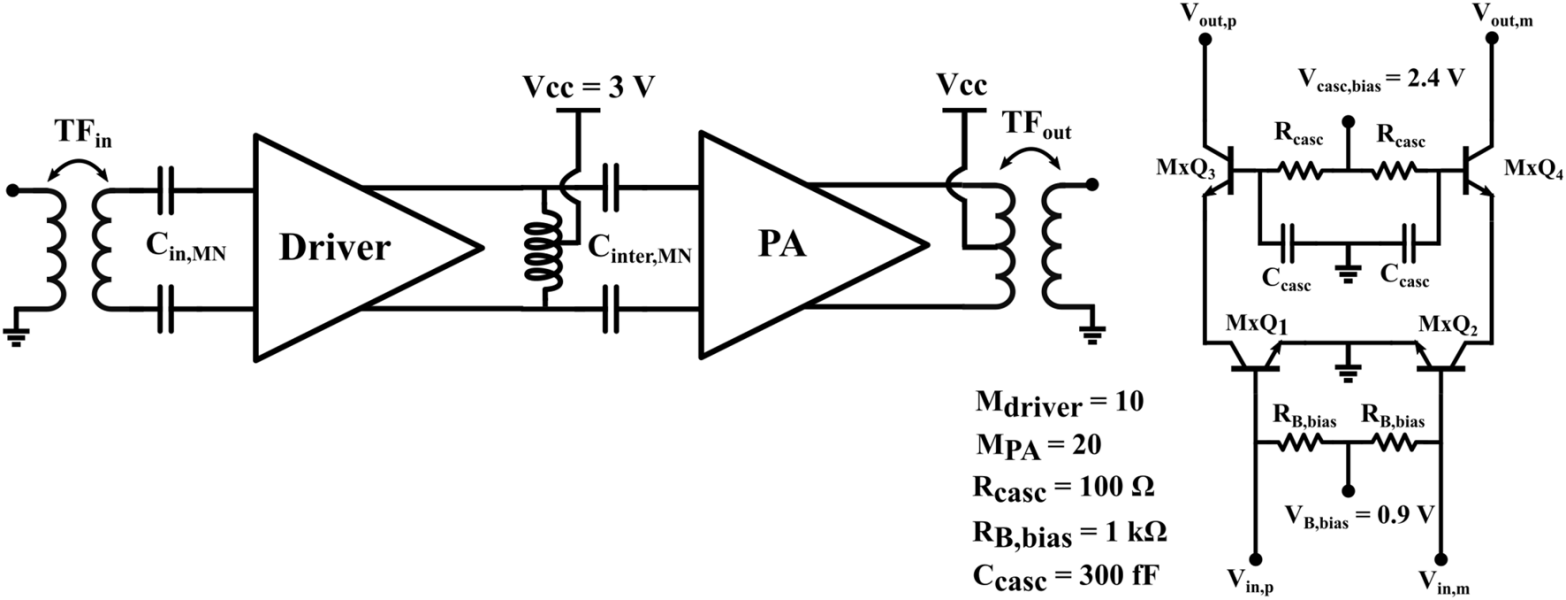
Schematic of the proposed power amplifier and internal schematic of a cascode amplifier stage.

**Figure 3:**
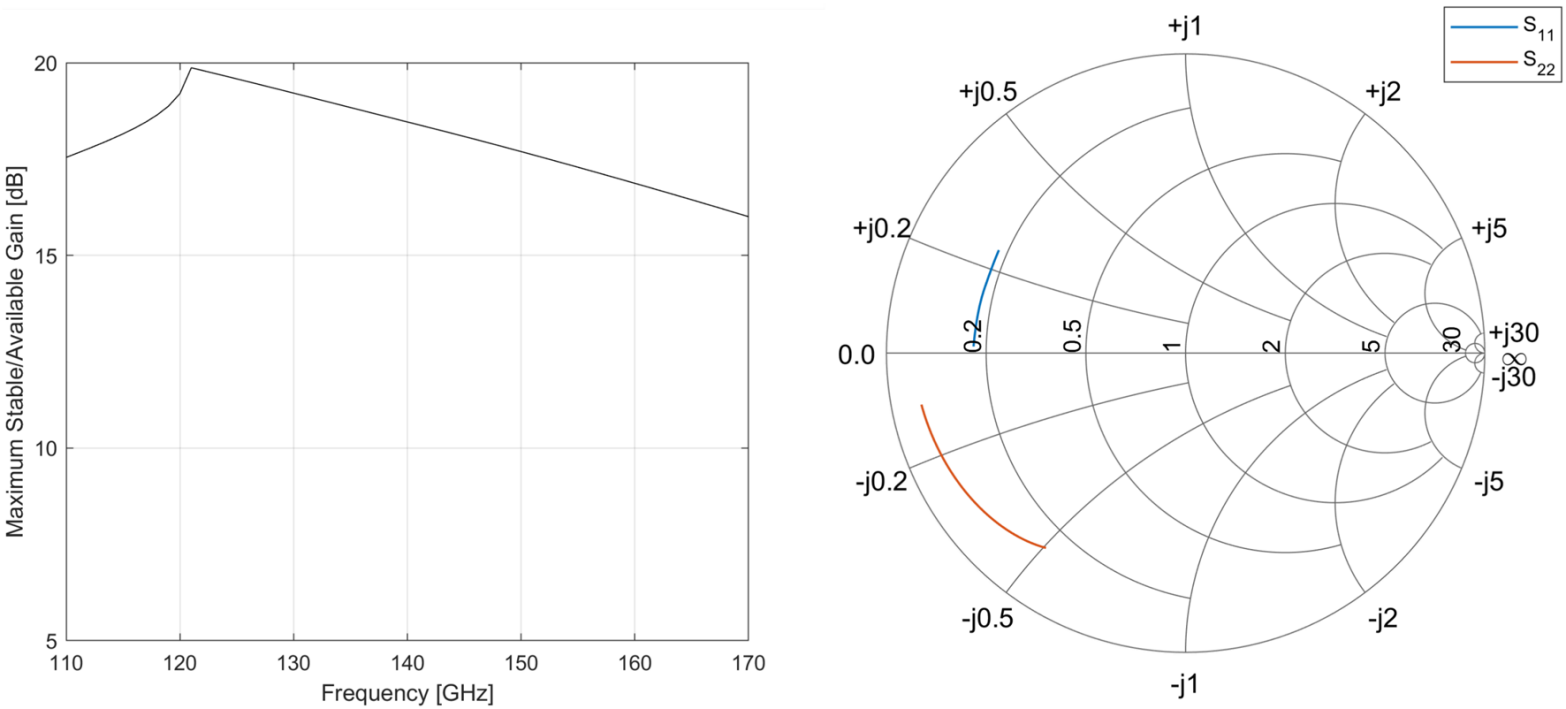
Co-simulated S-parameters of the cascode circuit.

The schematic of the proposed power amplifier is shown in Figure 2. The circuit consists of a driver stage and a power stage which are both biased in class A to maintain high gain and linearity. Both stages feature a similar pseudo-differential cascode topology. Using a cascode circuit has multiple advantages. First, a higher gain can be achieved than with common emitter (CE) or common base (CB) configurations. Co-simulating the schematic-level transistor model provided by the foundry combined with the EM-simulated layout of basic interconnection metallization, both the CE and CB configurations yield a maximum available gain (MAG) of around 8 dB. However, when looking at Figure 3, which showcases the MAG and the input/output reflection coefficients of the cascode circuit, we see that its performance is significantly higher, achieving a MAG of over 18 dB at ${140}\,\hbox {GHz}$. Note that the right part of the MAG curve depicts the maximum stable gain (MSG) as opposed to the MAG since the amplifier is not unconditionally stable. The loss required to stabilize the amplifier is handled through the inherently lossy matching circuits. This is in stark contrast with the simple CE and CB configurations which would need very low-loss matching networks to produce a useful amount of gain in this technology. The higher gain per stage also reduces the required number of stages to reach a suitable gain specification. Combined with the compactness of the topology, this significantly reduces the footprint of the final IC. In order to boost the gain of the cascode amplifier, an internal matching network is sometimes implemented in between the two transistors. Such a matching network can reduce the impedance mismatch between the output of the bottom CE transistor and the input of the top cascode transistor, essentially a CB amplifier, increasing the MAG. Using a tool such as the S-Parameter Probe component in Keysight ADS, the internal impedance levels at this node when looking into either side of the circuit can be obtained without disturbing its function. This indeed shows a discrepancy between both quantities. However, even when both are conjugately matched to eachother using a matching circuit, the increase in MAG is negligible considering the space needed for the implementation of such a matching circuit.

**Figure 4:**
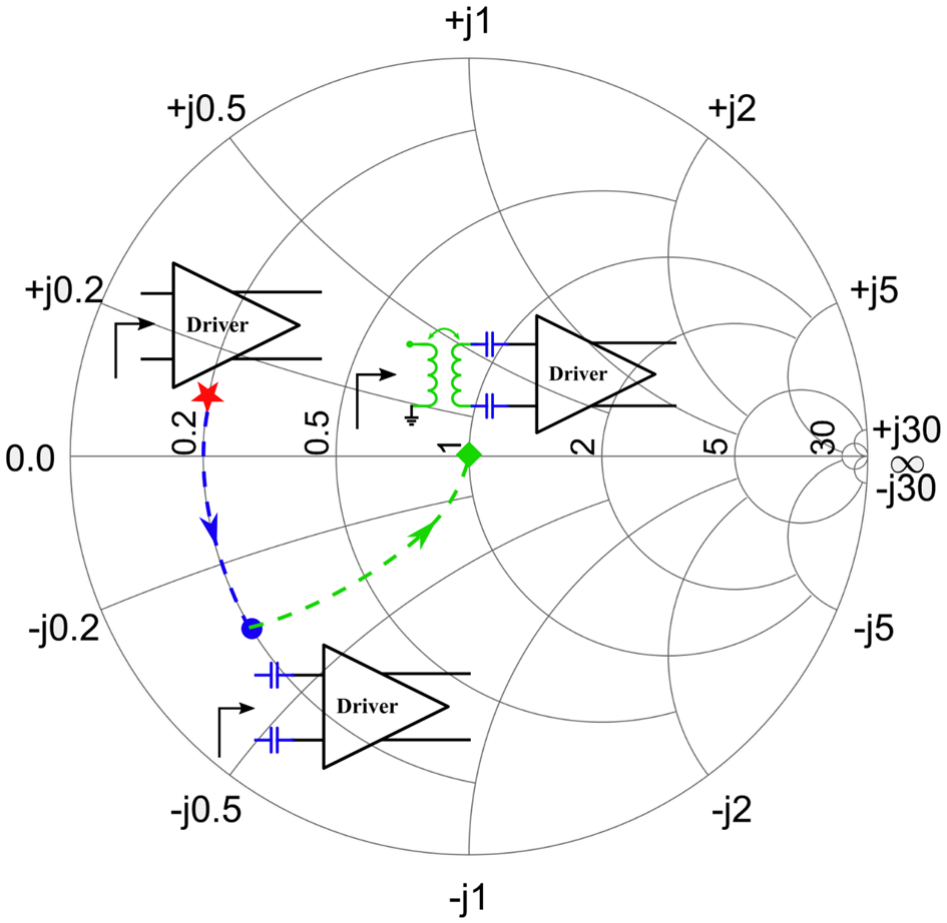
Matching topology used at the input of the driver stage.

The second major advantage of the cascode topology is its increased supply voltage as the voltage swing at the output of the amplifier is now divided over 2 devices, although not equally. The higher supply voltage allows for an in increased voltage swing at the amplifier’s output, which in turn yields a higher maximum output power. Lastly, the pseudo-differential character of the circuit signifies that while the core circuit operates differentially, the interfacing with the IC is done in a single-ended way. In this case, the single-ended to differential conversion is achieved through integrated input and output transformers operating as baluns, indicated as $TF_{in}$ and $TF_{out}$ on the schematic in Figure 2. Having a single-ended interface with the IC significantly simplifies measurements and also facilitates integration with other components. In this case, these baluns also operate as matching networks. However, a problem arises when trying to match the input of the driver or power stage to an inductive impedance such as a transformer. As shown in Figure 3, their input impedance is inductive due to the high frequency of operation, making it impossible to attain a good power match without additional components. The series capacitors $C_{in, MN}$ and $C_{inter, MN}$ are therefore added to the circuit to shift the input impedance back to the capacitive half-plane of the Smith chart. This impedance transformation is illustrated using the input matching network of the driver stage on the Smith chart in Figure 4.

In terms of sizing, we use 20-emitter devices in the power stage to obtain as much output power as possible while maintaining a realisable optimal load impedance. The driver stage is a scaled down version of the power stage, both in bias current and in the size of devices as the gain of the power stage lowers the output power requirement of any previous stages. Simulations were performed using the completed output stage to verify the impact of the driver stage output power on the complete amplifier’s compression point. This yielded that a driver stage with a scaling factor of 2 was sufficient to minimize its influence, giving rise to 10-emitter devices in the driver stage.

### Layout implementation

**Figure 5:**
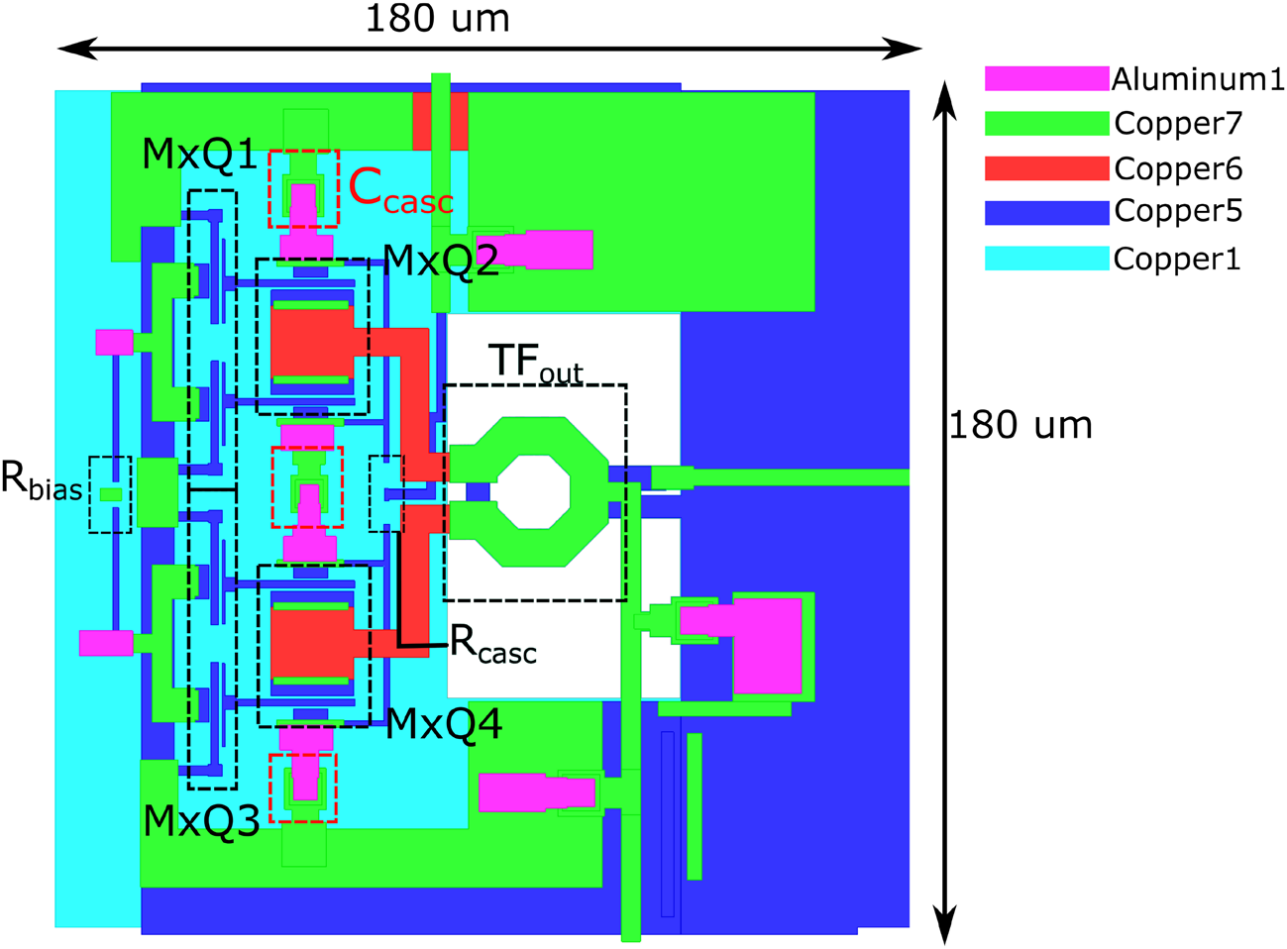
Top view of the layout of the PA stage.

Arguably the most critical step in a sub-THz RFIC design is translating the circuit to a layout as careless implementation will have a detrimental effect on the performance of the IC. When designing circuits operating at frequencies > 100 GHz, EM simulations of the entire RF core are an absolute necessity in the iterative design and optimization process but also as a means of verification. Figure 5 shows the layout of the PA’s output stage. Note the high degree of symmetry to ensure good differential operation and compact integration to minimize parasitics. As mentioned before, each transistor from the schematic in Figure 2 is implemented as 2 10-emitter devices to support higher output power.

Since this amplifier relies on a cascode topology operating at very high frequencies, great care needs to be taken to adequately decouple the base of every cascode transistor as it is well known that any parasitic inductance at this node can destabilize the amplifier^41^. Here, this potential issue is avoided by adding multiple decoupling stages to the relevant base nodes. First, high frequency decoupling is provided very close to the base of all cascode transistors using high-Q, high self-resonance frequency (SRF), low-capacitance MIM capacitors, indicated by $C_{casc}$ on the schematic in Figure 2 and the layout in Figure 5. A ${100}\,\Omega$ series resistor $R_{casc}$ is then added to dampen potential oscillations. This component also diminishes the influence of hard to model external components such as bondwires and DC sources on the impedance level seen at these base nodes, thereby increasing the predictability of the circuit. Finally, low-Q, high capacitance decoupling cells further away from the devices are added. This ensures an impedance that is low at high frequencies and well-modeled for lower frequencies at every potentially problematic node.

Taking this into account, an EM/circuit co-simulation of the PA stage’s core was carried out using Cadence EMX. This model was then used in a load-pull simulation to determine the PA’s optimum load impedance $Z_{L,opt}$. This yielded an optimum load impedance of $3.8 + 14j \Omega$. Notice that the resistive part of $Z_{L,opt}$ is rather small; this is a consequence of the high number of parallel devices in the output stage, meaning that the size of this stage is close to the upper limit for this technology at this frequency. The small resistive part of $Z_{L,opt}$ also implies that little parasitic resistance can be tolerated in the output matching network.

**Figure 6:**
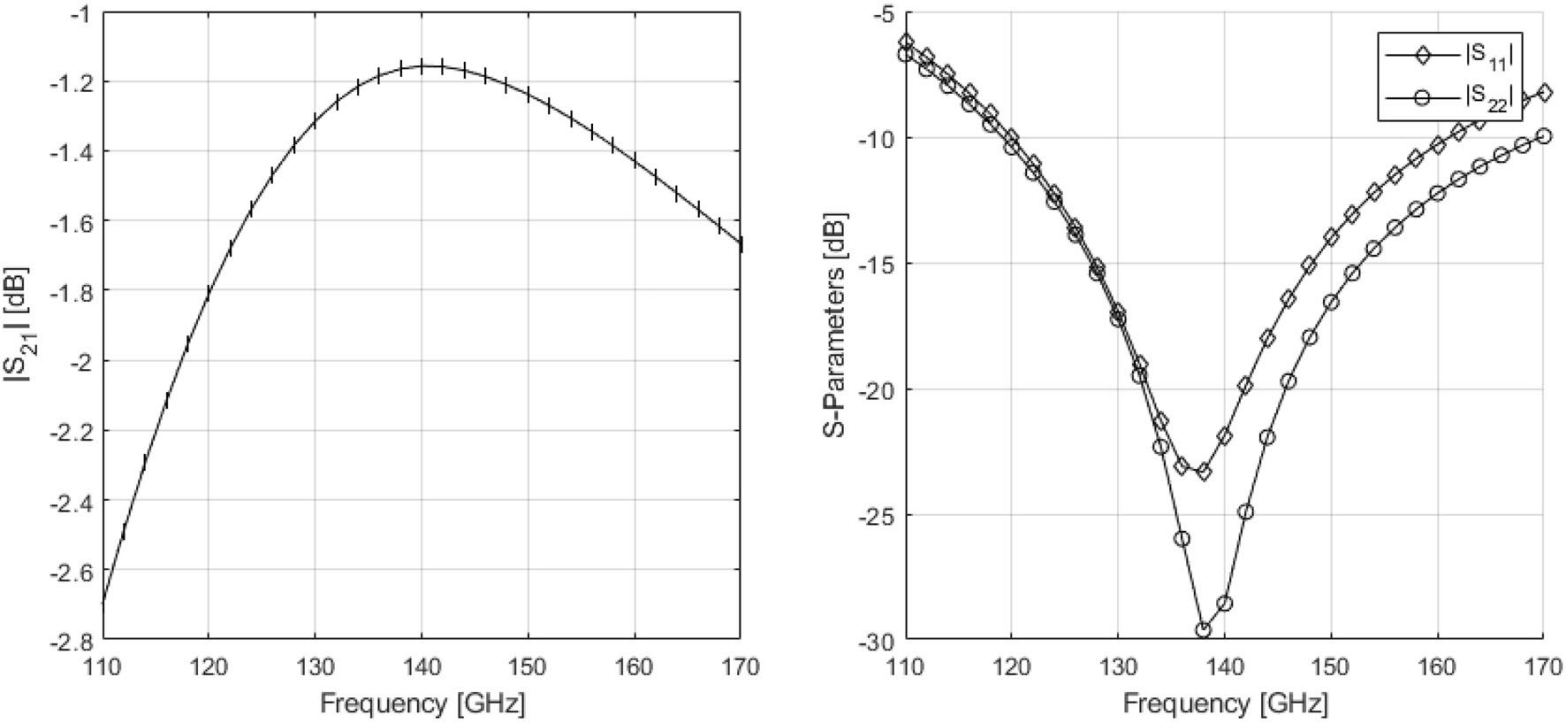
S-parameters of the optimized output transformer.

The design of the output matching network, a transformer in this case, is handled different from a classic matching network. Since the impedance transformation realized by the balun is fairly complex at these frequencies, it will be considered as a normal 2-port device that will be optimized for input/output matching as well as transmission. This means that during the design procedure, we consider the transformer’s simultaneously matched source and load reflection coefficients ($\Gamma _{S/L,SM}$) as well as its MAG, signifying the transformer’s inherent loss. The goal now becomes to design a transformer with $\Gamma _{S,SM} \leftrightarrow Z_{L,opt}^{*}$, $\Gamma _{L,SM} \leftrightarrow 50 \Omega$ and a MAG that is as high as possible. To achieve the low resistive impedance part at the input, the primary winding of the output balun is comprised of multiple, thick metal layers to minimize parasitic resistance. The output matching requirement initially poses a problem as a transformer is inherently an inductive device, meaning that $\Gamma _{L,SM}$ will always have a capacitive component unless the transformer is used exactly at its SRF, which would require a very large transformer. However, this issue can easily solved by adding a capacitor in parallel with the secondary windings, resonating out the inductive part. In practice, this capacitor can be absorbed in the output transmission line by slightly lowering its characteristic impedance. In order to increase matching between simulation and measurements, the transformer was encased with metallic sidewalls that are connected to the chip’s groundplane. Note that no ground shield underneath the transformer was used as the coupling to it increases the total loss by over a dB. The transformer was designed and optimized using Cadence EMX. Its S-parameters with respect to $Z_{L,opt}^{*}$ at the input and $50 \Omega$ at the output are presented in Figure 6. The transformer achieves a transmission of -1.1 dB and a return loss of more than 20 dB at both input and output.

### Measurement results

**Figure 7:**
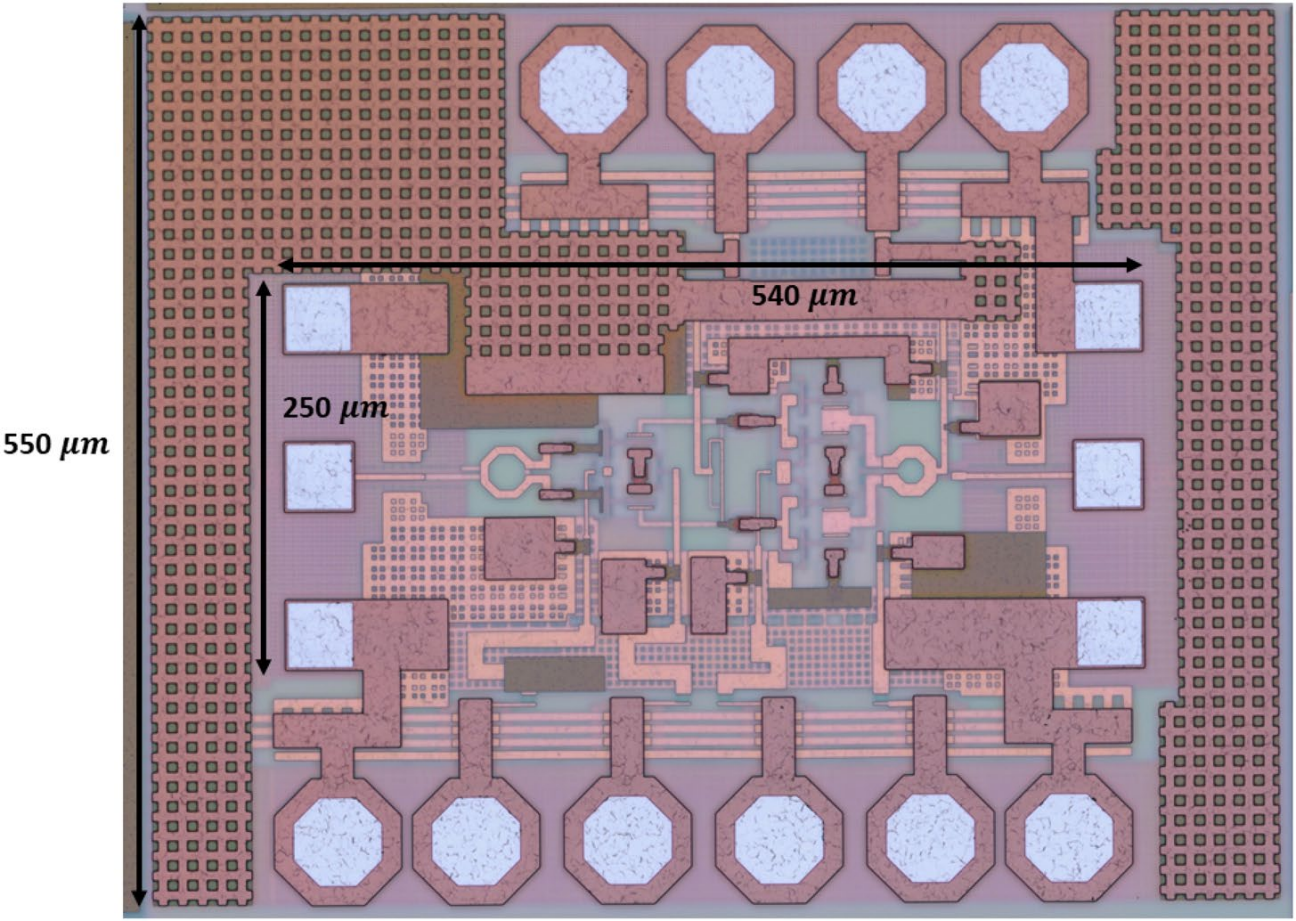
Micrograph of the fabricated chip.

**Figure 8:**
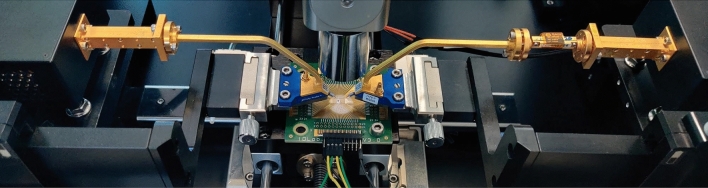
Measurement setup used to characterize the power amplifier IC in small signal and large signal regime.

The power amplifier IC is fabricated using the IHP SG13G2Cu process. Its micrograph is shown in Figure 7. The amplifier’s total area is 540×550 μm^2^, while the RF core only takes up 250 × 540μm^2^. The amplifier is first characterized in small-signal as well as in large-signal regime. Small-signal measurements are obtained via range extender-based S-parameter measurements and large-signal performance is assessed by performing a power sweep at the input of the amplifier and measuring transmission to the output. This allows us to extract the PA’s 1-dB compression point and its saturated output power. All of these measurements were performed via on-wafer probing using an MPI TS200-THZ Probing System and FormFactor Infinity waveguide probes. This measurement setup is shown in Figure 8. The waveguide probes are directly connected to VDI WR6.5 VNAX TxRx range extender modules which in turn are connected to a Keysight N5247B PNA-X. Using a GSG calibration susbtrate, an S-parameter calibration was performed up to the probe tips.

### Small-signal measurements

**Figure 9:**
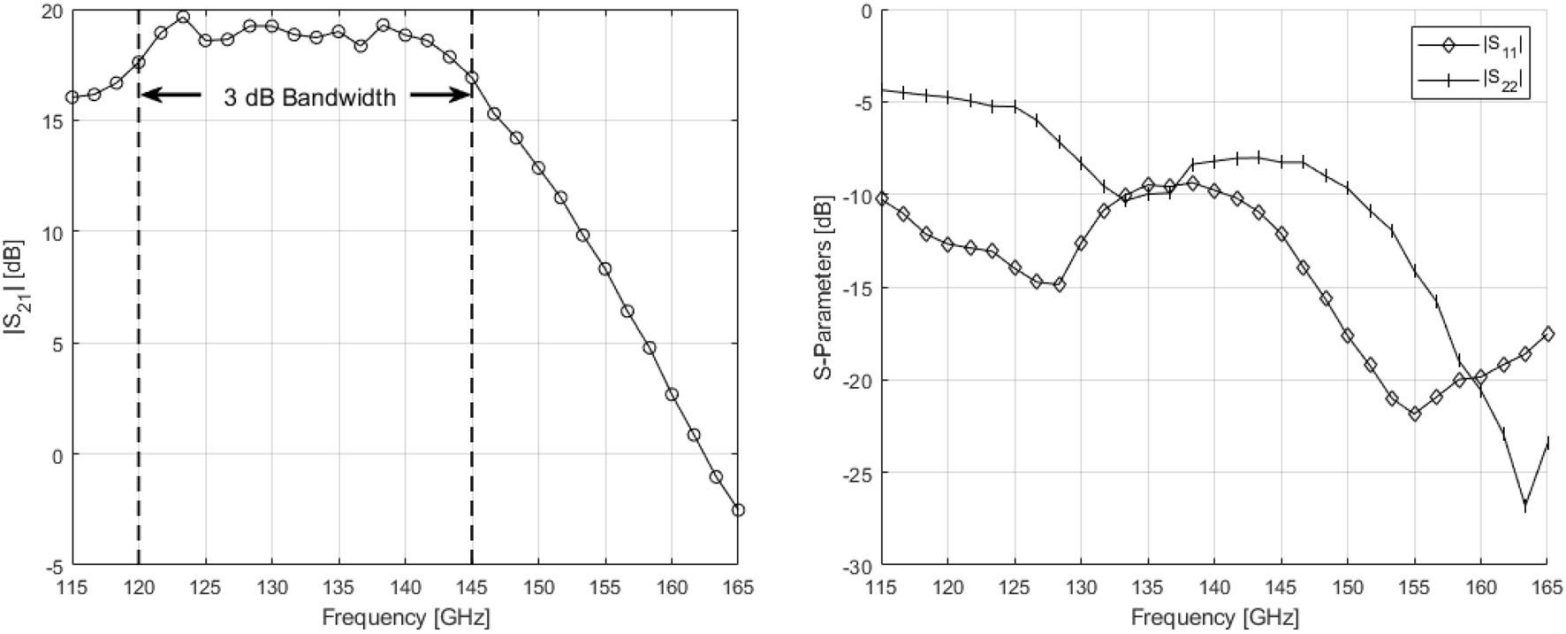
Small-Signal S-Parameter measurements of the power amplifier IC.

Figure 9 depicts the S-parameter measurement data. The amplifier exhibits a peak gain of approximately 20 dB with a 3 dB bandwidth of 25 GHz between 120 and 145 GHz. Additionally, the amplifier’s input reflection coefficient shows a -10dB bandwidth that encompasses almost the complete measurement range aside from a small peak around 135 GHz.

### Large-signal measurements

**Figure 10:**
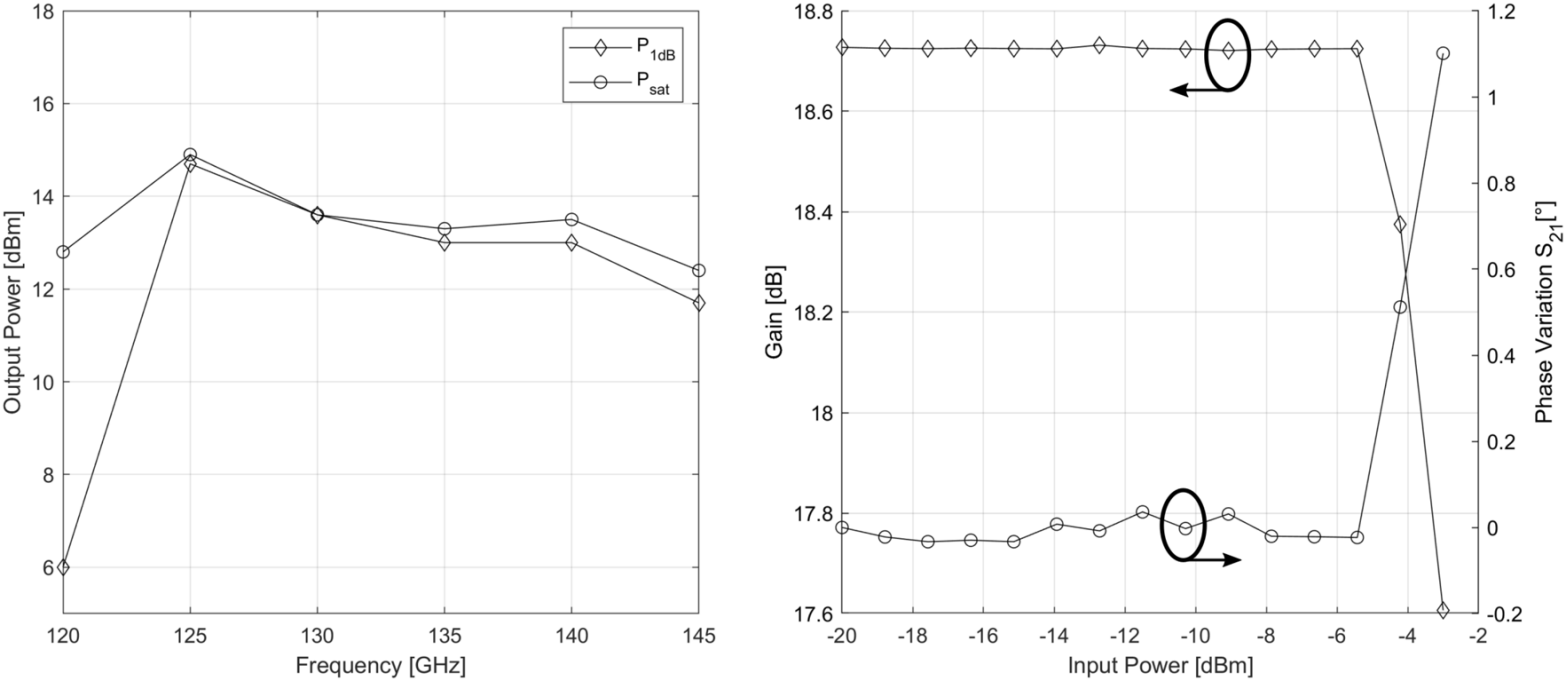
Large signal measurements of the power amplifier IC.

In order to perform large-signal/output power measurements, a source/receiver power calibration was performed up to the probe tips. Afterwards, the source power was swept and the output power and $S_{21}$ are measured at different frequencies from ${120}\,\hbox {GHz}$ to ${145}\,\hbox {GHz}$. Figure 10 depicts $P_{1dB}$ and $P_{sat}$ over frequency as well the AM-AM and AM-PM distortion at ${125}\,\hbox {GHz}$. The 1dB output compression point of the power amplifier peaks at ${125}\,\hbox {GHz}$ at 14.6 dBm while the highest measured output power is 15 dBm. It should be noted that due to the limitations of the measurement equipment, the PA could not be completely saturated. This results in a 6.5% power-added efficiency (PAE) at 1dB compression and a maximum PAE of 7%. Note that the low PAE is a direct consequence of not being able to saturate the PA as it is essentially still operating under backoff. It is also worthy to note that the thermal dissipation of these PAs will not be negligible when assembled into an array as any D-band PA’s low efficiency will give rise to a thermal power density of close to ${0.5}\,\hbox {W}/\hbox {mm}^{2}$. However, due to the system architecture’s design, this thermal power can easily be dissipated without disturbing the antenna elements.

Table 1 gives an overview of D-band power amplifiers in literature. Here we can see that this IC achieves state-of-the-art performance when compared to other SiGe implementations. Important to note is that many chips that achieve higher output power exploit power combining, which drastically increases IC size. This makes these chips unsuitable for application in $\frac{\lambda }{2}$-spaced phased arrays. Consequently, our implementation is currently the most compact implementation of a D-band power amplifier to achieve this level of output power with the closest contender only including the RF core footprint of 323 μm× 790 μm while exploiting a process technology that is not yet commercially available.

**Table 1:** Overview of state-of-the-art D-band power amplifiers in literature.

Ref.	Technology	Freq [GHz]	$\varvec{P_{1dB}}$ [dBm]	$\varvec{P_{sat}}$ [dBm]	Gain [dB]	3dB-BW [GHz]	$\varvec{PAE_{max}}$ [%]	Chip size [μm^2^]
This work	${130}\,\hbox {nm}$ SiGe	135	14.6	15	20	25	7	250 × 450^1^ 540 × 550
^42^	${90}\,\hbox {nm}$ SiGe	130	18.6*	21.9	18.2	10	12.5	1040 × 1640
^36^	${55}\,\hbox {nm}$ SiGe	135	18.5	19.3	22.4	25	13	323 × 790^1^
^8^	${22}\,\hbox {nm}$ CMOS-SOI	140	9.4	12.5	33.6	30	10.8	350 × 450^2^
^43^	${45}\,\hbox {nm}$ CMOS-SOI	135	13.5*	18.5	24.8	15	11	560 × 820^1^
^44^	${40}\,\hbox {nm}$ CMOS	140	10.7	14.8	20.3	17	8.9	540 × 623^1^
^39^	InP	130	22.3*	24	29.4	35.6	6	920 × 2050
^37^	InP	145	9*	12	17	20	4.5	1700 × 1900

$^{1}$ RF circuit core only, no bondpads included

$^{2}$ RF circuit core, bondpads included, estimated from micrograph

*Using power combining

### Modulation measurements

**Figure 11:**
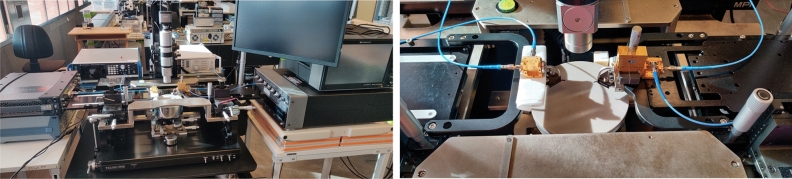
Measurement setup used for modulated signal transmission.

**Figure 12:**
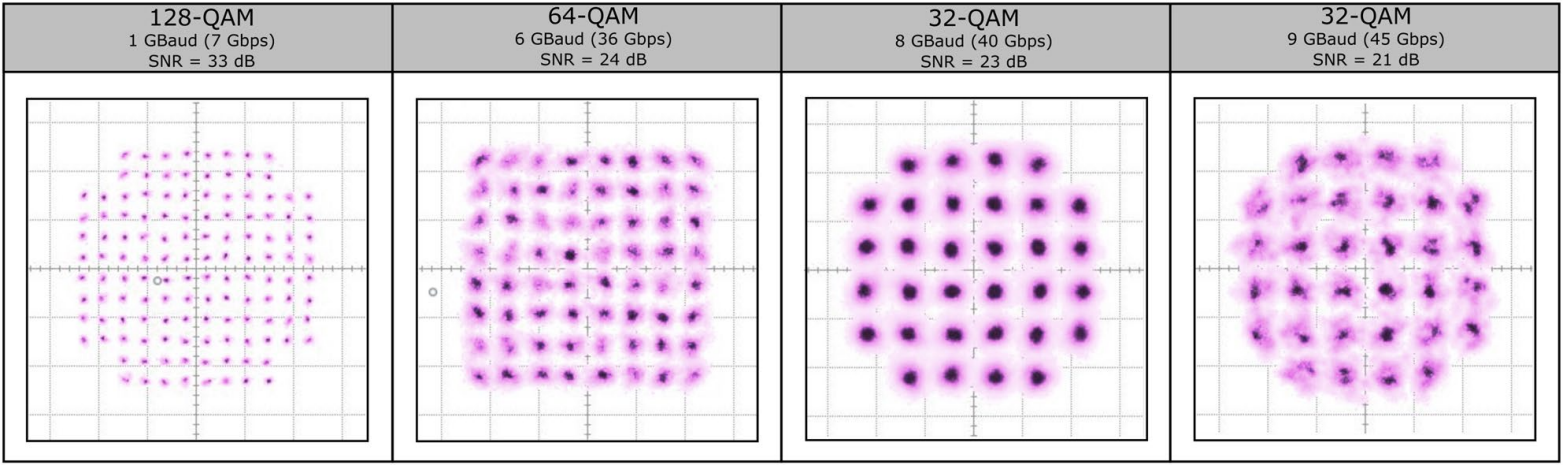
Constellation diagrams of transmitted modulated waveforms.

Finally, time domain measurements were performed on the PA through the transmission of modulated waveforms. This directly illustrates the PA’s performance for its intended use case. The modulated signal transmission setup is shown in Figure 11. All components are mounted on an MPI TS-150 THZ probe station to allow for interfacing with the device-under-test (DUT). Using a Keysight M8196A arbitrary waveform generator (AWG), a data signal is generated on an IF carrier at 15 GHz. This IF signal is then applied to a VDI WR6.5SHM sub-harmonic waveguide mixer. The LO signal is provided by a Rohde & Schwarz SMA100B signal generator. The upconverted RF signal is injected into the DUT through a GGB PICOPROBE Model 170 WR-6 waveguide probe. Using a similar probe at the output of the DUT, the amplified signal is transferred into a Flann Microwave WR-6 variable waveguide attenuator to reduce the signal power to a safe input level for the second VDI subharmonic downconversion mixer. This second mixer uses an LO signal provided by an Anritsu MG3697C signal generator that has been locked to the first LO source. The downconverted IF signal is then analyzed and demodulated by a Teledyne LeCroy 10-65-Zi-A real-time oscilloscope (RTO) using its vector signal analysis (VSA) software. Note that no pre-distortion or equalization is applied to the signal. The resulting constellation diagrams can be seen in Figure 12. This shows that the PA is able to transmit highly complex constellations such as 64-QAM or 128-QAM while maintaining a very high SNR. Moreover, a maximum data rate of 45 Gbps is achieved using a 9 GBaud, 32-QAM format, all while maintaining an SNR larger than 20 dB.

## Conclusion

This paper proposed a novel concept for wideband, 2D steerable, opto-electronic phased antenna arrays operating at 140 GHz. It presented the envisioned system architecture and its advantage over the current state-of-the-art, leveraging optical components to implement both low-loss signal distribution and TTD beamforming while using compact active opto-electronic antenna modules to perform optical-electric conversion, amplification and radiation. Moreover, the design, development, and characterization of a compact power amplifier chip in SiGe technology is presented, a key component for the realization of such photonic-enabled transmitter modules. The power amplifier IC features a gain of 20 dB over a bandwidth of ${25}\,\hbox {GHz}$ between ${120}\,\hbox {GHz}$ and ${145}\,\hbox {GHz}$ and shows a 1dB output power of 14.6 dBm with a saturated output power of 15 dBm. On top of this, modulated signal measurements show that the IC is able to transmit data at speeds up to 45 Gbps. All of this is achieved in a footprint of 540 μm × 550 μm , making it one of the fastest and most powerful D-band power amplifiers for its footprint, thereby paving the way for wideband, fully 2D-steerable opto-electronic antenna arrays.

## Data Availability

The datasets used and/or analysed during the current study available from the corresponding author on reasonable request.
